# Supplementary material for: Dopant-Free Hole Transport Materials with a Long Alkyl Chain for Stable Perovskite Solar Cells
Source: Nanomaterials (Basel). 2019 Jun 28;9(7):935. doi: 10.3390/nano9070935 (PMC6669455; doi:10.3390/nano9070935)
Supplement: Supplementary file 1 [file nanomaterials-09-00935-s001.pdf]

nanomaterials

Supplementary Materials

Dopant-Free Hole Transport Materials with Long Alkyl Chain for Stable Perovskite Solar Cells

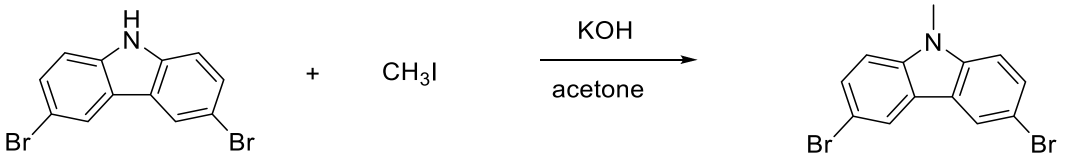

3,6-dibromo-9-methyl-*9H*-carbazole (1)

Carbazole (2 g, 6.15 mmol) was added in 20 mL acetone. Then KOH (1.38 g, 24.62 mmol) was put into the solution. Iodomethane (1.05 g, 7.38 mmol) was added and stirred overnight. The mixture was washed with water, and extracted with CH_2Cl_2. The organic phase dried over anhydrous Na_2SO_4.After removal of the solvent , The residue was purified by column chromatography with petroleum ether to afford the compound **1** as a light white soild. 1H NMR (300 MHz, $CDCl_3$) δ 8.14 (d, J = 1.7 Hz, 2H), 7.59 (dd, J = 8.7, 1.8 Hz, 2H), 7.27 (d, J = 8.6 Hz, 2H), 3.82 (s, 2H).

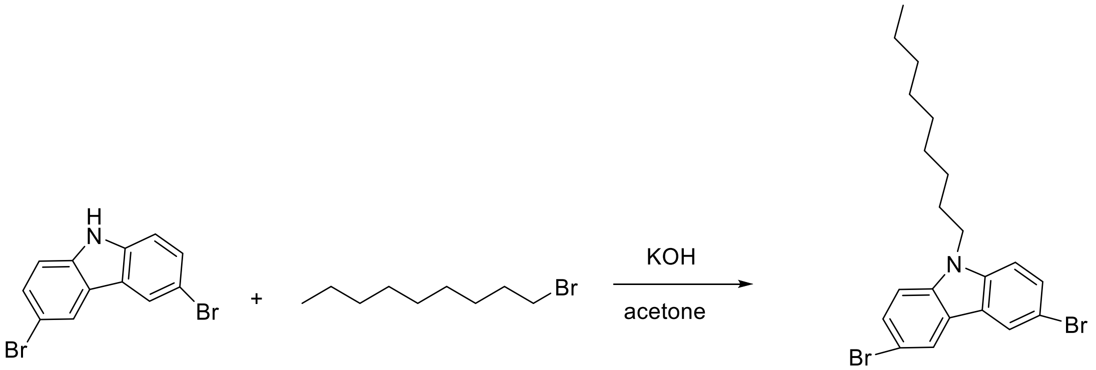

3,6-dibromo-9-nonyl-*9H*-carbazole (2)

Carbazole (2 g, 6.15 mmol) was added in 20 mL acetone. Then KOH (1.38 g, 24.62 mmol) was put into the solution.1-Bromooctane (1.53 g, 7.38 mmol) was added and stirred overnight. The mixture was washed with water, and extracted with CH_2Cl_2. The organic phase dried over anhydrous Na_2SO_4.After removal of the solvent , The residue was purified by column chromatography with petroleum ether to afford the compound **2** as a light yellow oil. 1H NMR (300 MHz, $CDCl_3$) δ 8.12 (d, J = 1.5 Hz, 2H), 7.53 (dd, J = 8.7, 1.7 Hz, 2H), 7.24 (m, 2H), 4.21 (t, J = 7.2 Hz, 2H), 1.80 (m, 2H), 1.21 (s, 12H), 0.86 (d, J = 6.2 Hz, 3H).

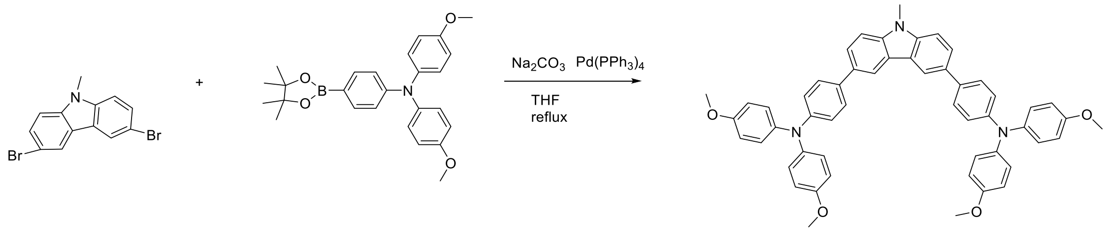

4,4'-(9-methyl-9*H*-carbazole-3,6-diyl)bis(*N*,*N*-bis(4-methoxyphenyl)aniline)

Compound **4** (300 mg, 0.88 mmol), N,N-bis(4-Methoxyphenyl)-4-(4,4,5,5-tetraMethyl-1,3,2-dioxaborolan-2-yl)-BenzenaMine (1.15 g, 2.65 mmol) and $Pd(PPh_3)_4$ (153 mg, 0.13 mmol) were placed in a Shrek bottle. Tetrahydrofuran (20 mL with molecular

sieves) and Na_2CO_3 solution (4 mL, 2.0 M) were added. The mixture was bubbling for 20 minutes with Nitrogen. The mixture was refluxed for 24 h. After cooling to room temperature, The mixture was washed with water, and extracted with CH_2Cl_2. After removal of the solvent, **CZTPA-1** (0.48 g, 70%) was obtained by column chromatography on silica gel using petroleum ether/dichloromethane (4:1). 1H NMR (400 MHz, $CDCl_3$) δ 8.30 (s, 2H), 7.70 (d, J = 8.5 Hz, 2H), 7.56 (d, J = 8.3 Hz, 4H), 7.43 (d, J = 8.5 Hz, 2H), 7.11 (s, 12H), 6.86 (d, J = 8.7 Hz, 8H), 3.85 (d, J = 22.6 Hz, 15H). 13C NMR (101 MHz, CDCl3) δ 155.70, 147.32, 141.10, 140.57, 132.13, 128.20, 126.98, 124.91, 123.41, 121.49, 118.22, 114.67, 108.74, 77.37, 77.06, 76.74, 55.53, 29.31. MALDI-TOF MS: m/z=787.34 $[M+H]^+$, calcd. for $C_{61}H_{61}N_3O_4$: 787.05.

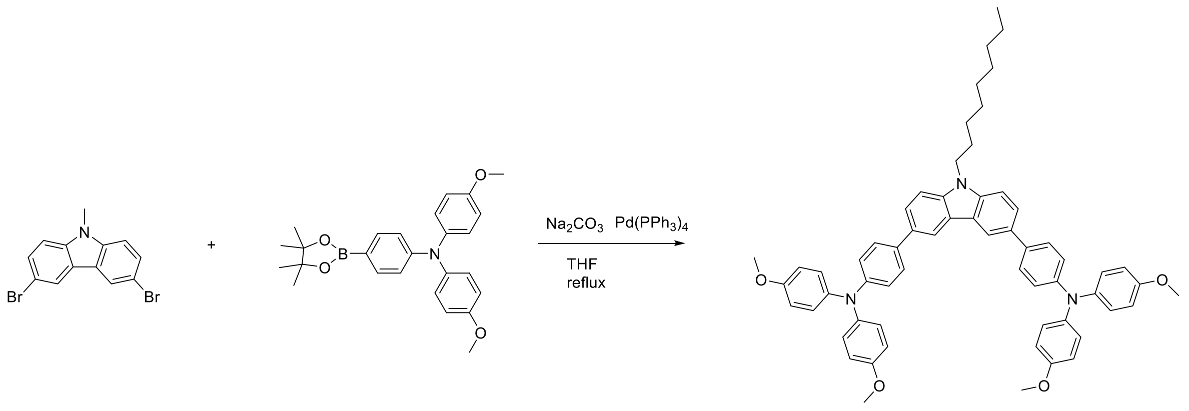

4,4'-(9-nonyl-9*H*-carbazole-3,6-diyl)bis(*N*,*N*-bis(4-methoxyphenyl)aniline)

Compound **1** (300 mg, 0.67 mmol), N,N-bis(4-Methoxyphenyl)-4-(4,4,5,5-tetraMethyl-1,3,2-dioxaborolan-2-yl)-BenzenaMine (0.86 g, 1.99 mmol) and Pd(PPh_3)$_4$ (115 mg, 0.1 mmol) were placed in a Shrek bottle. Tetrahydrofuran (20 mL with molecular sieves) and Na_2CO_3 solution (4 mL, 2.0 M) were added. The mixture was bubbling for 20 minutes with Nitrogen. The mixture was refluxed for 24 h. After cooling to room temperature, The mixture was washed with water, and extracted with CH_2Cl_2. After removal of the solvent,. **CZTPA-2** (0.4 g, 68%) was obtained by column chromatography on silica gel using petroleum ether/dichloromethane (4:1). 1H NMR (400 MHz, CDCl3) δ 8.29 (s, 2H), 7.68 (d, J = 8.5 Hz, 2H), 7.54 (d, J = 8.5 Hz, 4H), 7.43 (d, J = 8.6 Hz, 2H), 7.09 (dd, J = 23.4, 8.6 Hz, 12H), 6.86 (d, J = 8.8 Hz, 8H), 4.31 (t, J = 6.8 Hz, 2H), 3.82 (s, 12H), 1.90 (m, 2H), 1.35 (m, 12H), 0.87 (t, J = 6.7 Hz, 3H). 13C NMR (101 MHz, CDCl3) δ 155.66, 141.21, 127.66, 126.35, 121.43, 114.67, 77.37, 77.06, 76.74, 55.54, 29.42. MALDI-TOF MS: m/z=899.91 $[M+H]^+$, calcd. for $C_{61}H_{61}N_3O_4$: 899.47.

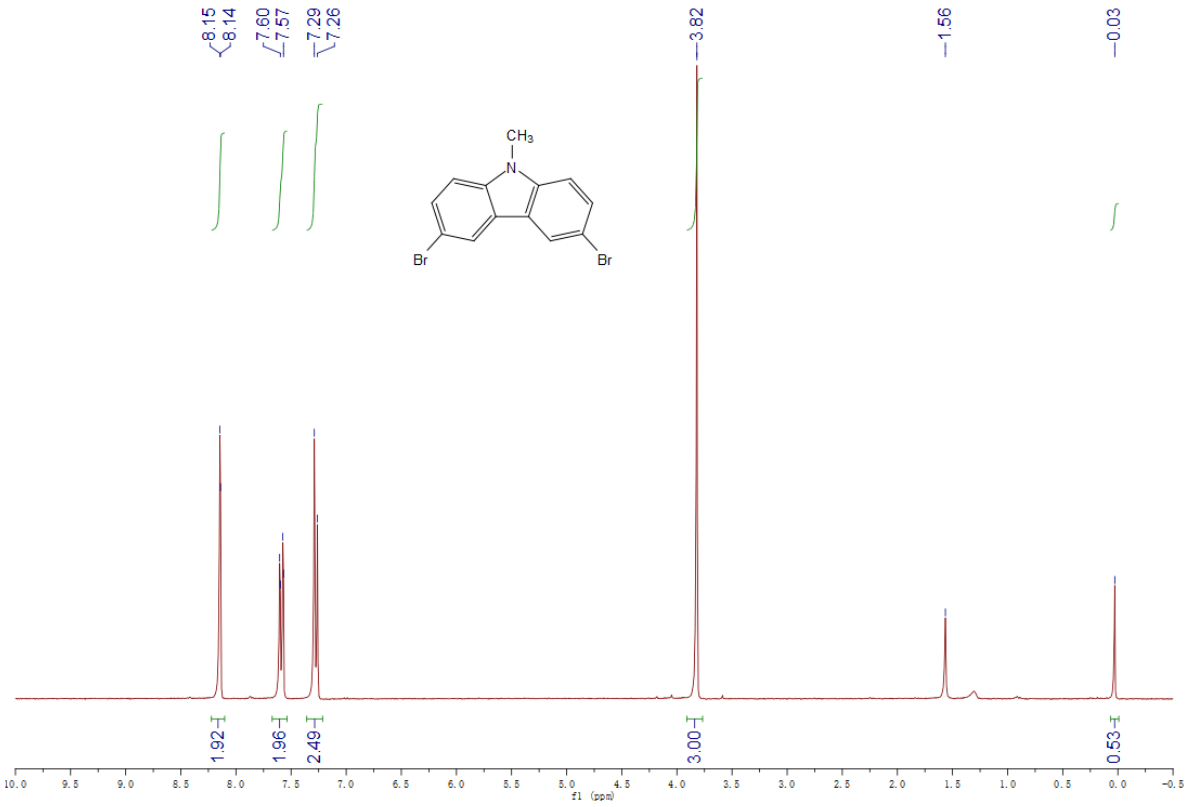

Figure S1. ^{1}H NMR spectrum for compound **1**

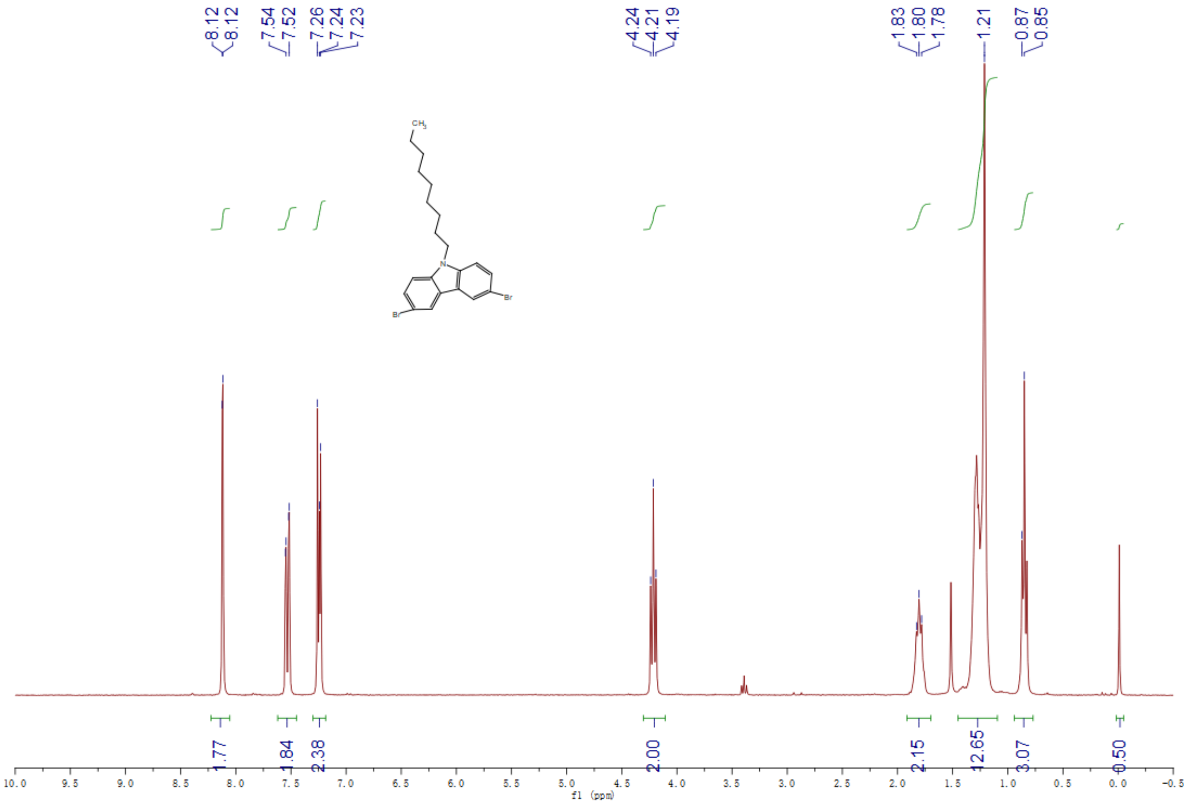

Figure S2. ^{1}H NMR spectrum for compound **2**.

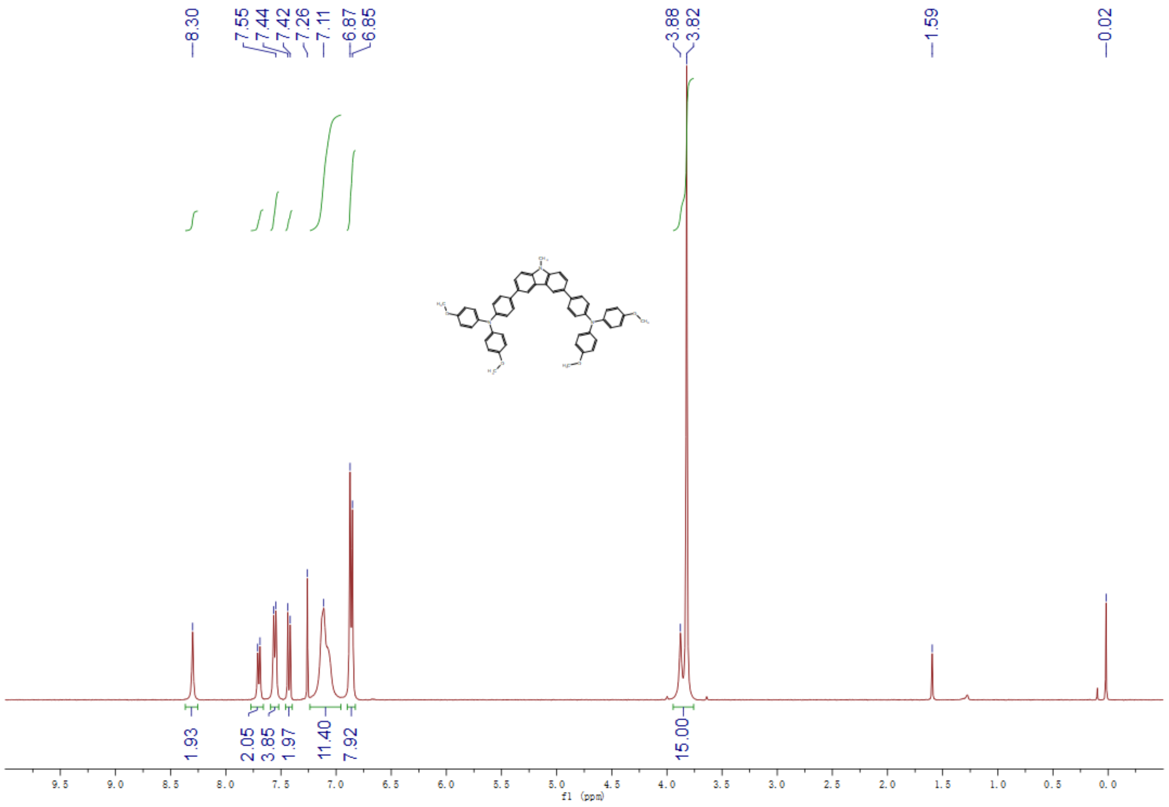

Figure S3. ¹H NMR spectrum for CZTPA-1.

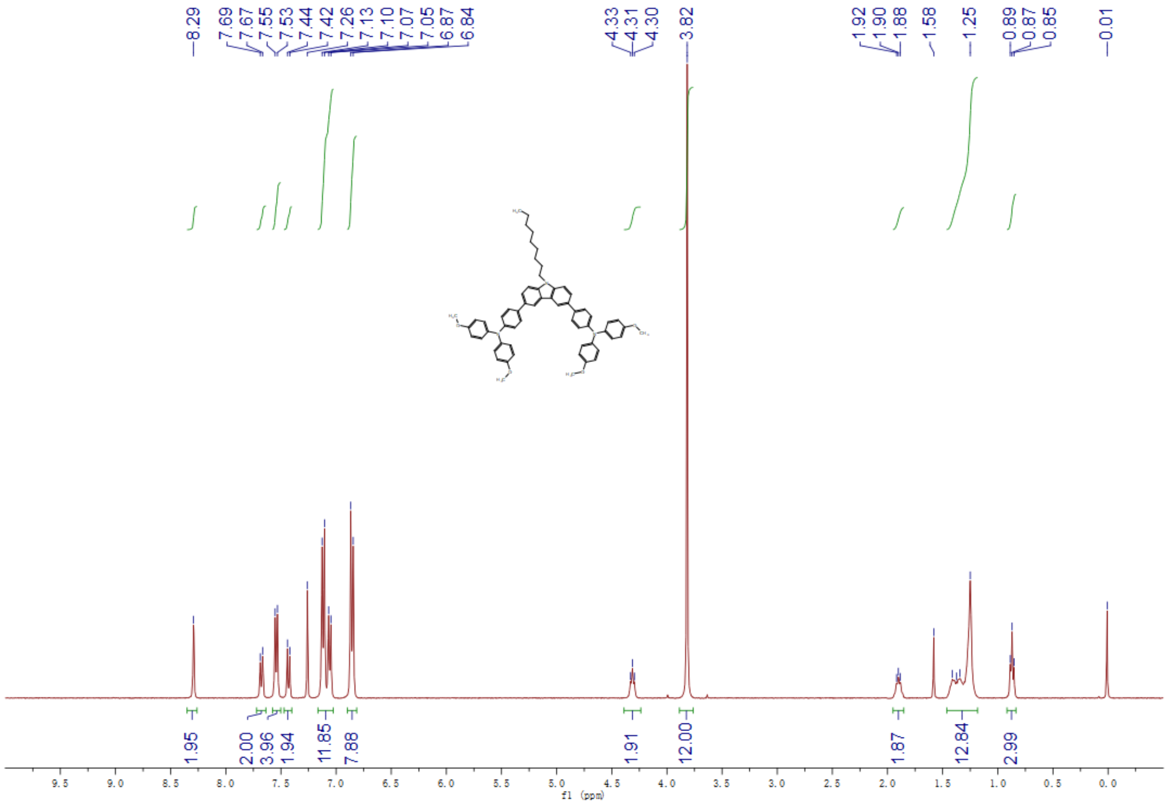

Figure S4. ¹H NMR spectrum for CZTPA-2.

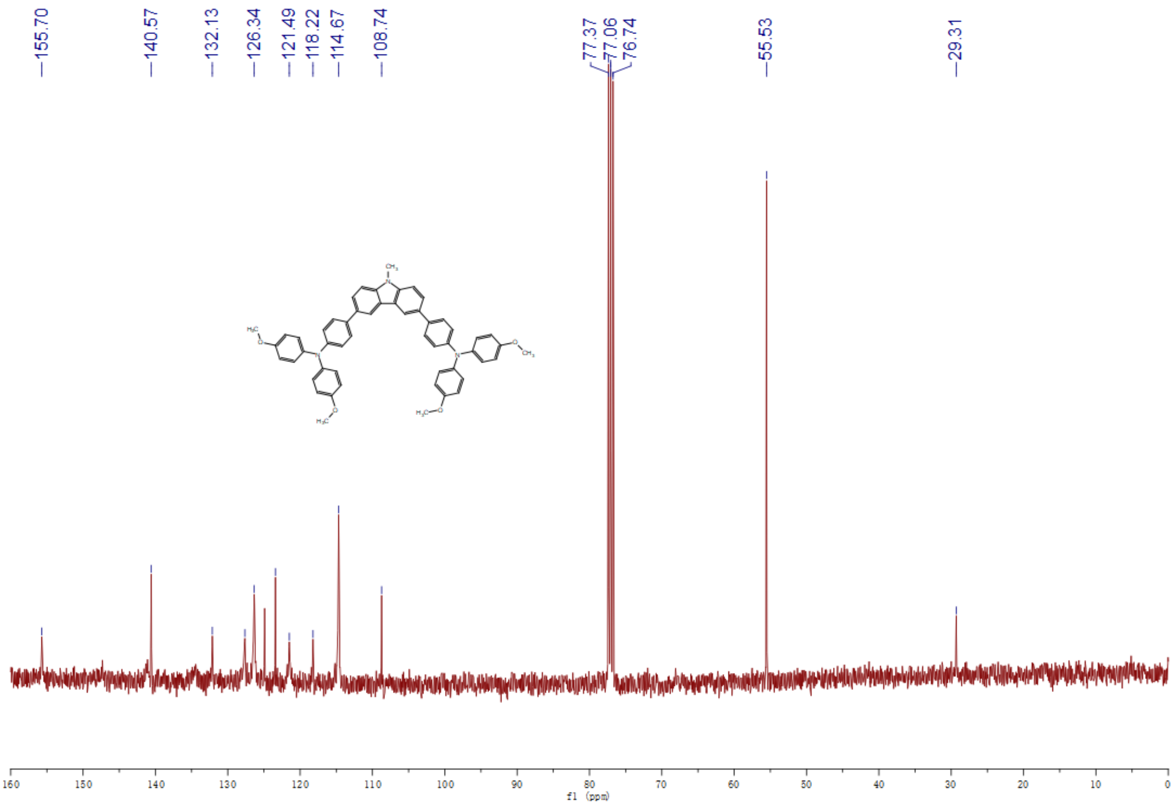

Figure S5. ^{13}C NMR spectrum for CZTPA-1.

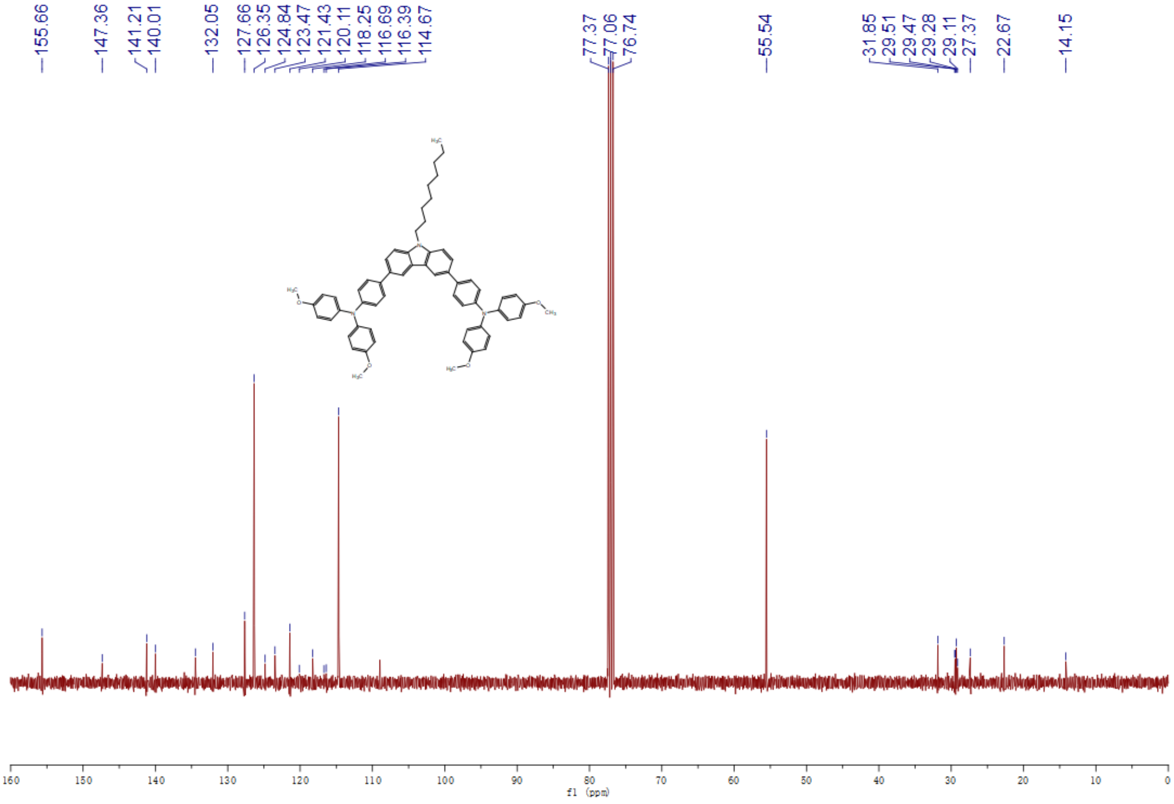

Figure S6. ^{13}C NMR spectrum for CZTPA-2.

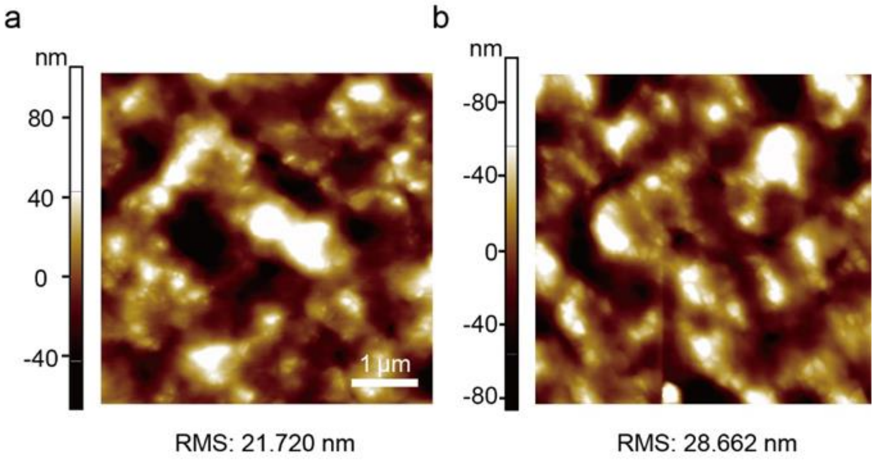

Figure S7. AFM images (5 µm x 5 µm) of CZTPA-2 (a) and CZTPA-1 (b) films.
